# Micro-CT results exhibit ovules enclosed in the ovaries of *Nanjinganthus*

**DOI:** 10.1038/s41598-022-27334-0

**Published:** 2023-01-09

**Authors:** Qiang Fu, Yemao Hou, Pengfei Yin, José Bienvenido Diez, Mike Pole, Manuel García-Ávila, Xin Wang

**Affiliations:** 1 State Key Laboratory of Palaeobiology and Stratigraphy, Nanjing Institute of Geology and Palaeontology and CAS Center for Excellence in Life and Paleoenvironment, Chinese Academy of Sciences, Nanjing, 210008 China; 2 Key Laboratory of Vertebrate Evolution and Human Origins of Chinese Academy of Sciences, Institute of Vertebrate Paleontology and Paleoanthropology, CAS Center for Excellence in Life and Paleoenvironment, Chinese Academy of Sciences, Beijing, 100044 China; 3 Departamento de Xeociencias Mariñas e Ordenación do Territorio, Universidade de Vigo, 36200 Vigo, Spain; 4 Centro de Investigación Mariña, Universidade de Vigo (CIM-UVIGO), 36200 Vigo, Spain; 5 Queensland Herbarium, Mount Coot-Tha Road, Toowong, QLD 4066 Australia

**Keywords:** Evolution, Plant sciences

## Abstract

The Early Jurassic angiosperm *Nanjinganthus* has triggered a heated debate among botanists, partially due to the fact that the enclosed ovules were visible to naked eyes only when the ovary is broken but not visible when the closed ovary is intact. Although traditional technologies cannot confirm the existence of ovules in a closed ovary, newly available Micro-CT can non-destructively reveal internal features of fossil plants. Here, we performed Micro-CT observations on three dimensionally preserved coalified compressions of *Nanjinganthus*. Our outcomes corroborate the conclusion given by Fu et al., namely, that *Nanjinganthus* is an Early Jurassic angiosperm.

## Introduction

The discovery of the Early Jurassic angiosperm *Nanjinganthus*^1^ triggered a heated botanical debate among botanists^2–6^. When they claimed the existence of two or more ovules within an ovary of *Nanjinganthus*^1,6^, there was a dilemma for Fu et al.: in a single specimen of *Nanjinganthus* flower, the ovule is either visible when the ovary is broken or is invisible when the ovary is intact and closed, but no ovule is visible in an intact closed ovary, as traditional technologies do not allow to demonstrate both ovules and the intact enclosing ovary in a single specimen. Technically, if these two features (ovules and closed ovary) cannot be proven in a single specimen, the angiospermous affinity of *Nanjinganthus* remains speculative. This leaves Fu et al.^1,6^ vulnerable to criticism. The application of Micro-CT technology enables us to non-destructively reveal the internal features that are otherwise hard or impossible to show in fossil plants^7^. To expel this final doubt over *Nanjinganthus*, we performed Micro-CT observations on three-dimensionally preserved coalified compressions of *Nanjinganthus*. The outcomes corroborate the conclusion that *Nanjinganthus* is an Early Jurassic angiosperm.

## Results

**Figure 1:**
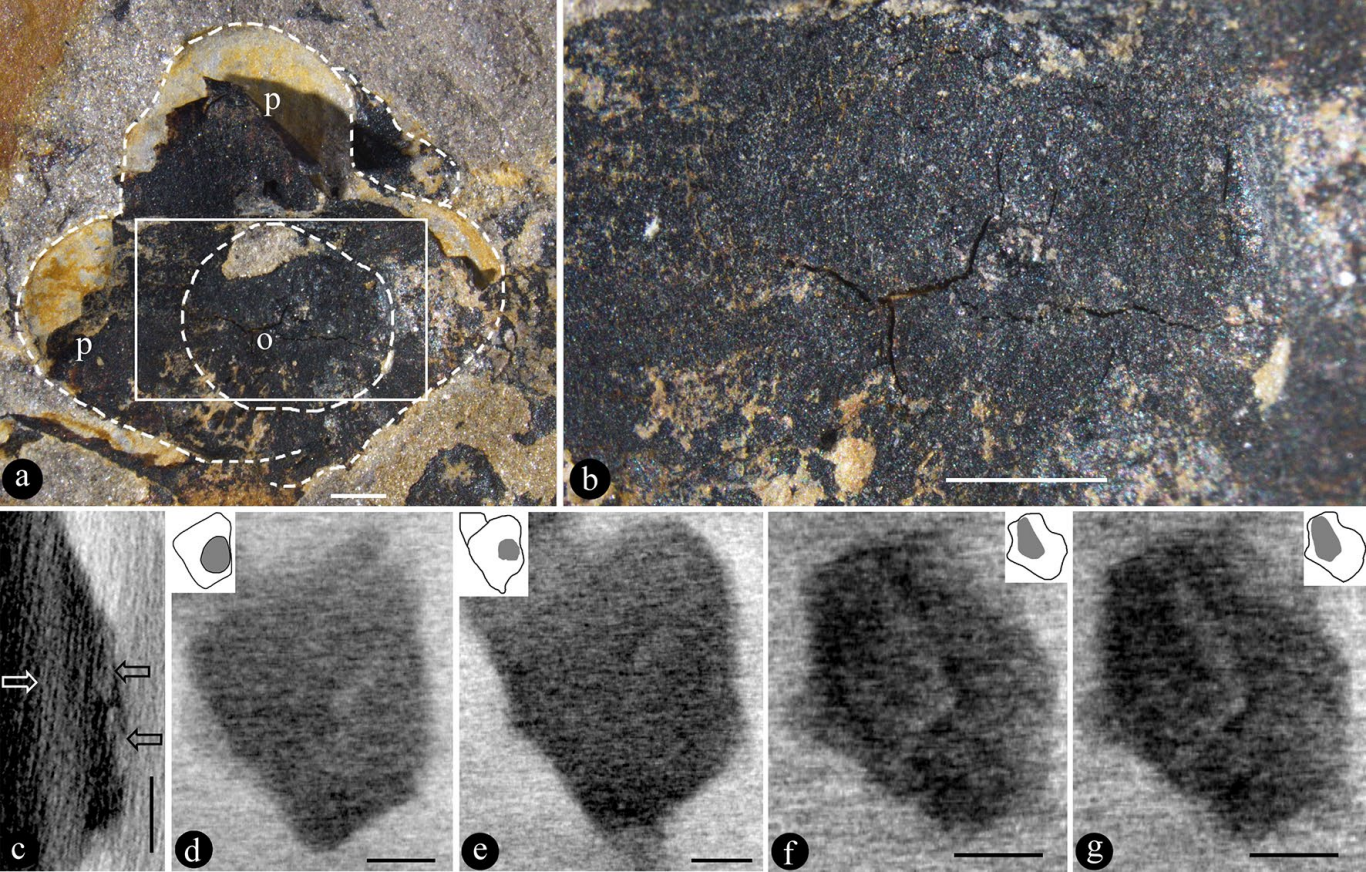
*Nanjinganthus dendrostyla* and its ovules within the ovary. PB22279. All scale bar = 1 mm. (**a**) Top-down view of the coalified compression, with an ovary (o) in the centre and one of the petals (p) peeling off from the sediments. The outlines of petals and ovary are marked with broken lines. Reproduced from Fu et al.^6^. (**b**) Integral ovary roof, with cracks due to preservation. (**c**–**g**) are micro-CT virtual sections. Reproduced from Fu et al.^6^. (**c**) Vertical section showing two ovules (black arrows) covered by the ovary roof (white arrow). (**d**,**e**) Two transverse sections showing one oval ovule within the ovary, refer to the inset, in which an ovule is grey in colour. (**f**,**g**) Two transverse sections showing another truncate-cuneate ovule in the ovary, refer to the inset, in which an ovule is grey in color.

PB22279 is a coalified flower compressed top-down (Fig. 1a). The diameter of the flower is approximately 8.6 mm, while the diameter of the ovary is approximately 3.5 mm (Fig. 1a). The epigynous flower bears petals and sepals on the upper rim of the ovary (Fig. 1a). The original ovary roof is integral, sealing the ovary completely, and only vaguely visible in the Micro-CT virtual section of the flower (Fig. 1a–c). The ovules are eclipsed by the ovary roof (Fig. 1a,b). Micro-CT observation results exhibit that there are at least two ovules within the ovary (Fig. 1c–g). The ovules vary in size, form, and orientation (Fig. 1c–g). One ovule is oval, 2.22 × 1.95 mm (Fig. 1d,e), while the other ovule is truncate-cuneate, 2.0 × 1.24 mm (Fig. 1f,g). The ovules are 0.22 to 0.27 mm in thickness (Fig. 1c). The order of occurrence of ovules, ovary, and petals in a video agreed with that the flower is epigynous (Supplementary Video V1, V2).

**Figure 2:**
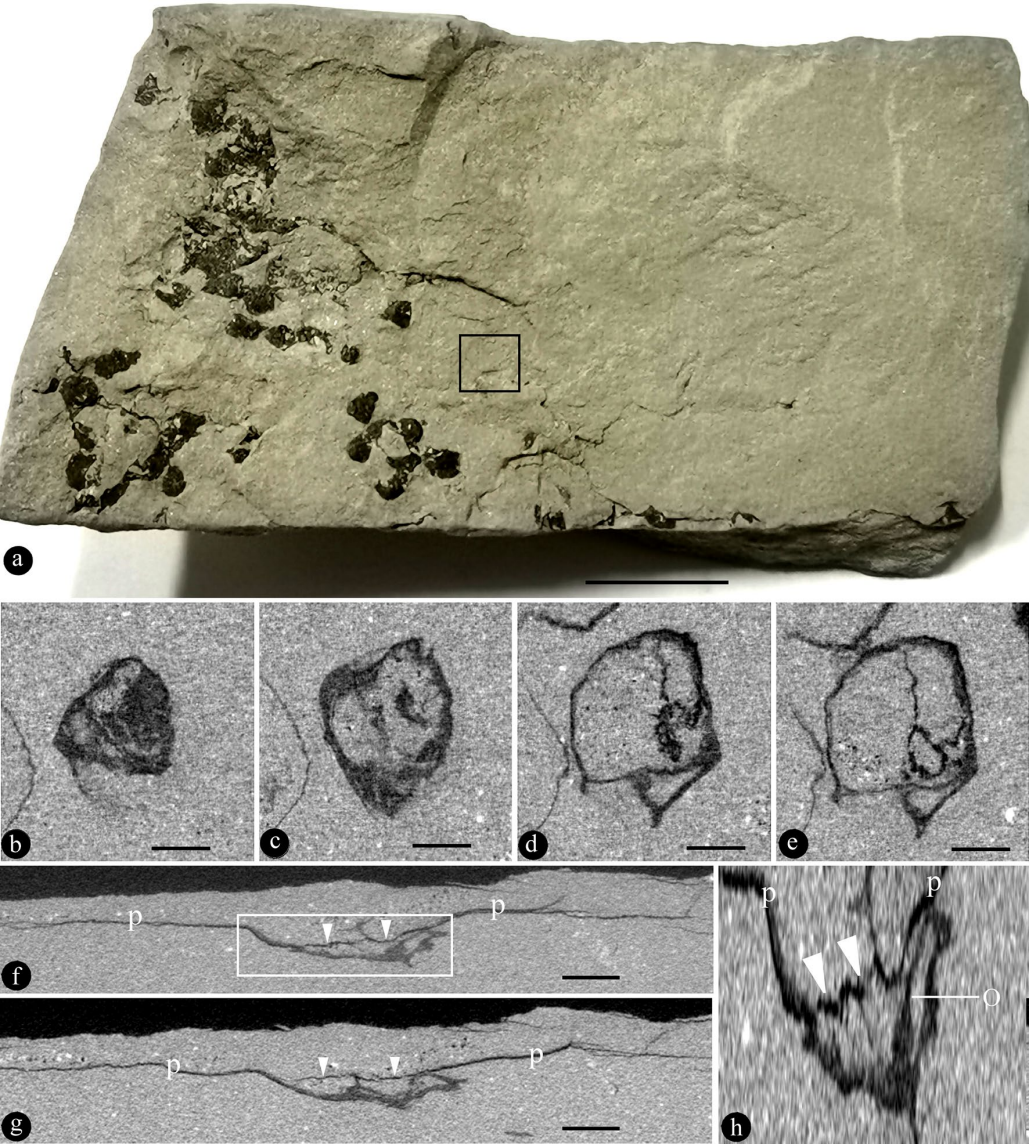
*Nanjinganthus dendrostyla* and its ovules within the ovary. PB180516. All scale bars = 1 mm, except annotated. (**a**) Several coalified compressions embedded in siltstone. Scale bar = 1 cm. (**b**–**e**) Virtual cross sections, in an ascending order, of the ovary of a flower fully embedded (invisible, in the rectangle in **a**) in the specimen shown in (**a**). (**f**,**g**) Two virtual vertical sections of the flower shown in (**b–e**), showing an ovary with an ovary roof (triangles) and petals (p). (**h**) A detailed view of the rectangular region in (**f**), showing a funipendulous ovule (o) attached to the inner wall of the ovary that has a roof (triangles) and petals (p). To make it easy to observe, the figure is vertically stretched by 700%.

PB180516 has multiple coalified flowers cumulated in the sediment (Fig. 2a). The flower focused on in this study is fully embedded in the sediment and thus invisible to naked eyes (Fig. 2b–h). The flower is preserved three-dimensionally, with an ovary including bowl-formed basal part and an ovary roof (Fig. 2b–h). The diameter of the ovary is 2.5–3.4 mm. On the top of the ovary is a thin layered ovary roof sealing the ovary, and around the ovary are petals (Fig. 2f–h). At least two ovules are recognizable within the ovary (Fig. 2b–h). In a virtual vertical section, an ovule can be seen attached to the side wall of the ovary through a funiculus (Figs. 2f,h, 4; Supplementary Video V3, V4).

**Figure 3:**
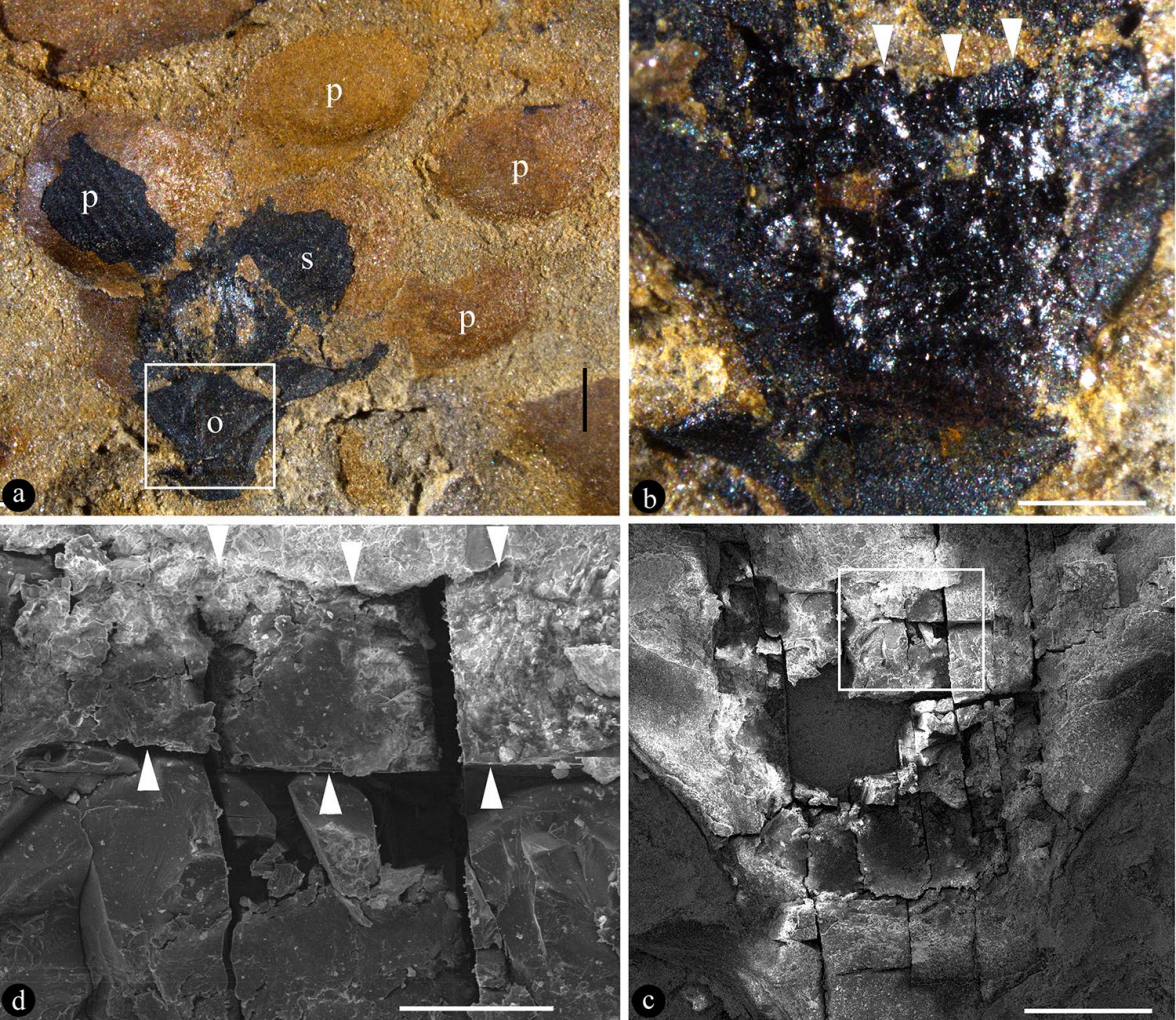
*Nanjinganthus dendrostyla*. PB22281. (**a**) A coalified laterally compressed flower with ovary (o), sepal (s), and petals (p), embedded in siltstone. Reproduced from Fu et al.^1^. Scale bar = 1 mm. (**b**) Detailed view of the basal portion of the flower shown in (**a**), showing clear border (triangles) between the sediment and ovary roof, exposed through dégagement. Scale bar = 0.5 mm. (**c**) SEM view of the portion shown in (**b**). Scale bar = 0.5 mm. (**d**) Detailed view of the rectangular region in (**c**), showing the ovary roof (between triangles) secluding the ovary from the exterior. Scale bar = 0.1 mm.

PB22281 is a laterally compressed coalified flower embedded in the sediment (Fig. 3a). Through dégagement, the peripheral portion of the ovary in foreground is removed, exposing the details near the ovary roof (Fig. 3b–d). The ovary roof is some flatly domed, making the ovary secluded (Fig. 3b–d). The ovary roof has smooth outer and inner surfaces, 122 μm thick, across the top of the ovary (Fig. 3b–d). The absence of sediment in the ovary suggests that the ovary is completely closed by the ovary roof (Fig. 3b).

## Discussions

**Figure 4:**
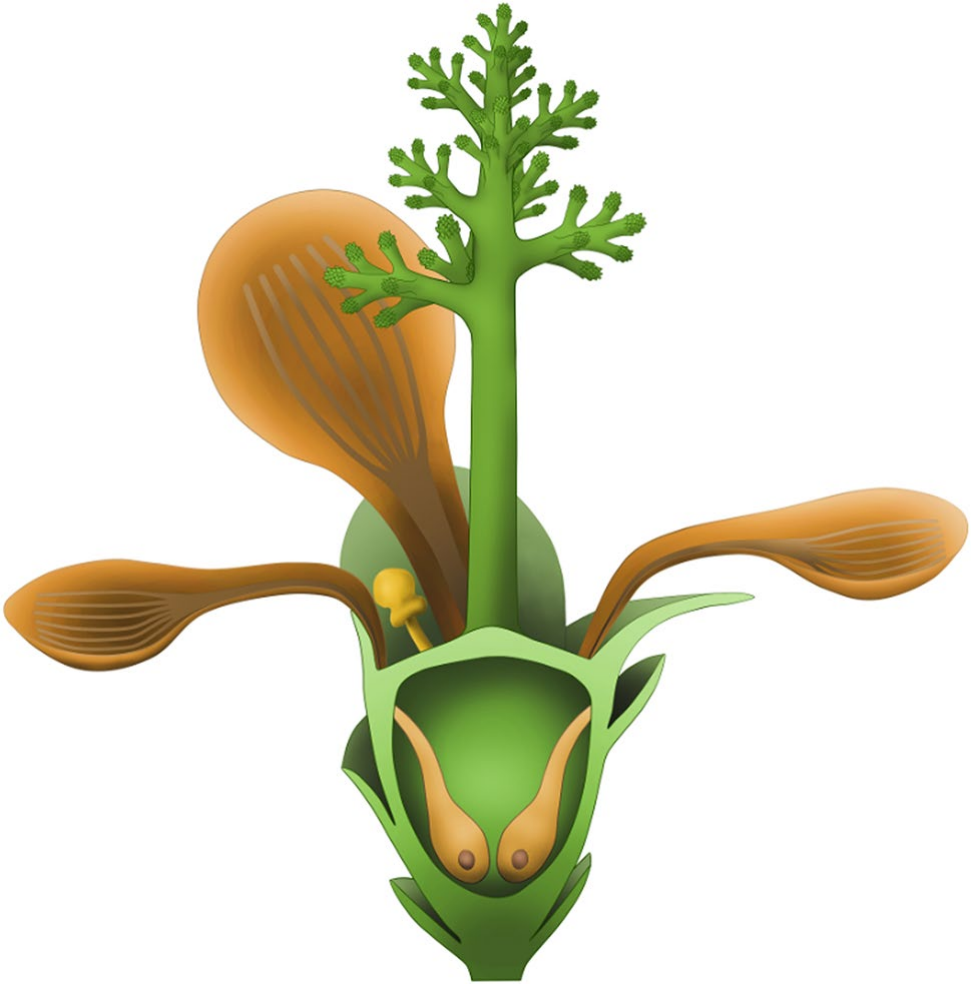
Updated reconstruction of *Nanjinganthus dendrostyla*. Note that the configuration of the ovule is slightly modified according to the new information revealed by Micro-CT.

It is obvious that *Nanjinganthus* is a reproductive organ, not a vegetative organ. Among all known reproductive organs, there are microspores, sporangia *sensu stricto*, and accessary foliar structures (e.g., bracts, sepals, petals, and involucrum). The ovules in the ovary of *Nanjinganthus* are of millimetric dimensions, 2.22 × 1.95 mm and 2.0 × 1.24 mm, and three dimensional (Fig. 1d–g). The forms and dimensions of these ovules and their connection to the ovary wall by a funiculus (Figs. 2f,h, 4) distinguish them from all accessory foliar structures frequently seen in reproductive organs. The dimensions of the ovules are much bigger than those of a microspore, which are usually not greater than 0.2 mm in any dimension. Sporangia *sensu stricto* are rarely known completely enclosed in a structure in any plants, with rare exceptions in *Marsilea* (which is morphologically distinct from *Nanjinganthus*, however). In contrast, the ovules in *Nanjinganthus* are enclosed, eliminating the possibility of these ovules being sporangia *sensu stricto*. These comparisons exclude microspore, sporangia, and accessory foliar structures from our consideration, leaving only two alternatives, an ovule/megaspore. The occurrence of a funiculus (the connection to the ovary wall) of the ovule (Fig. 2f, h) excludes megaspores from further consideration, as a megaspore (at least a mature one) is not connected to a mother plant. The variation of ovule forms seen in a single ovary of *Nanjinganthus* implies that the ovules may be asynchronous in development, a phenomenon frequently seen in extant angiosperms^8^.

The lack of consensus on the criterion for identifying fossil angiosperms has caused controversies in the study of early angiosperms. For example, Herendeen et al.^9^, Sokoloff et al.^2^, and Bateman^4^ have advanced mutually conflicting criteria for fossil angiosperms: Herendeen et al.^9^ put several characters as “unique angiosperm features”, Sokoloff et al.^2^ appeared to focus on pentamery of flowers, while Bateman^4^ preferred double fertilization and closed carpel. It became especially embarrassing when a reader finds that Bateman is a member of Sokoloff et al. and these two conflicting publications^2,4^ were online almost at the same time. Interestingly, either of these three criteria, if adopted, would annihilate almost all fossil angiosperms (including those published by the authors themselves), implying their inapplicability^6^. After a systematic survey of pollination in conifers, Tomlinson and Takaso^10^ found that some conifers do seclude their seeds (although such a seclusion occurs only after pollination), therefore “angiospermy” is not a feature unique of angiosperms, instead “ovules enclosed before pollination” draws a clear demarcation between gymnosperms and angiosperms. This criterion was adopted by Fu et al.^1,6^ and thus applied to determine that *Nanjinganthus* was an early angiosperm.

In surface view, the ovary roof is intact and integral in PB22279 (Fig. 1a,b), suggesting a closed ovary. Although obvious to naked eyes, the ovary roof in PB22279 is only vaguely observed in Micro-CT rendering. Similarly, ovary roof is seen as a thin seam in PB180516. This may be attributed to the top-down compression during the fossilization and the flowers in both specimens are similarly oriented and compressed. This explanation becomes more plausible when a laterally compressed flower is observed. As seen in Fig. 3a–d, the thickness of the ovary roof in PB22281 is less affected by the lateral compression during fossilization. Therefore, we can measure the thickness of the ovary roof, which is much thicker and more conspicuous than in PB22279 and PB180516. Although there are cracks in the ovary roof (Figs. 1b, 3c,d), these cracks can be attributed to artefacts from desiccation. The lack of sediments in the ovary (Fig. 3b) favours the integrity of the ovary roof. Taking all together, it is reasonable to say that the original ovary roof of *Nanjinganthus* is integral, and it secludes the ovary completely from the exterior.

Although Fu et al.^1,6^ demonstrated that the ovules were in the ovary in *Nanjinganthus* and they applied an over-strict criterion of angiosperms (it may lead some botanists to incorrectly place some angiosperms into gymnosperms), their argument is imperfect: (1) their conclusion was based on a comparison among over two hundred specimens, and (2) the ovules were demonstrated only in broken ovaries^1^. There is thus a paradox about *Nanjinganthus*: any ovules demonstrated are in broken ovaries, and no ovule is shown inside an intact ovary, then no one knows whether there really are ovules in an intact ovary of *Nanjinganthus*, since ovules and closed ovary, as two features, have never been demonstrated in the same specimen of *Nanjinganthus* hitherto. Thanks to the inventive Micro-CT technology, we can now demonstrate both these features (ovules and closed ovary) in PB22279 (Fig. 1a–g) and PB180516 (Fig. 2a–h), expelling this last doubt over *Nanjinganthus*. The in-ovary position of the ovules (Figs. 1d–g) suggests that angio-ovuly occurs in *Nanjinganthus*, satisfying the above over-strict criterion for angiosperms.

The early age (Early Jurassic) of *Nanjinganthus* is in line with increasing other fossil evidence^11–20^ as well as molecular studies and phylogenetic analyses^21–27^ suggesting an earlier origin of angiosperms (in the Jurassic and even the Triassic). It is time to update our knowledge about the early history of angiosperms.

### STAR methods

The materials studied here include two specimens that have been published previously in Fu et al.^1,6^ as well as one new specimen (Fig. 2). These specimens are from the same locality and the information of fossil locality, stratigraphy, and age is available in Fu et al. (2018). Details of the fossils were observed and photographed using a Nikon SMZ1500 stereomicroscope equipped with a Digital Sight DS-Fi1 camera. Two specimens (PB22279, PB180516) were scanned using a GE v|tome|x m300&180 micro-computed-tomography scanner (GE Measurement & Control Solutions, Wunstorf, Germany), housed at the Key Laboratory of Vertebrate Evolution and Human Origins of Chinese Academy of Sciences, Beijing, China. The data set has a resolution of 23.298 µm and the scan was carried out at 120 kV and 150 µA. One frame per projection was acquired by a timing of 2000 ms for a total of 2500 projections. One specimen (PB22281) was observed using a MAIA3 TESCAN SEM housed at the Nanjing Institute of Geology and Palaeontology, Nanjing, China. All images were recorded in TIFF or JPEG format, the videos were saved in avi format, all figures organized together using a Photoshop 7.0 for publication.

## Supplementary Information

Supplementary Information 1.

Supplementary Video 1.

Supplementary Video 2.

Supplementary Video 3.

Supplementary Video 4.

## Data Availability

All data analyzed during this study are included in this published article and its supplementary information files.
