# Supplementary material for: Micro-CT results exhibit ovules enclosed in the ovaries of Nanjinganthus
Source: Sci Rep. 2023 Jan 9;13:426. doi: 10.1038/s41598-022-27334-0 (PMC9829905; doi:10.1038/s41598-022-27334-0)
Supplement: Supplementary file 1 — Supplementary Information 1. [file 41598_2022_27334_MOESM1_ESM.docx]

**Supplementary**

V1. Video showing vertical sections of PB22279.

V2. Video showing cross sections of PB22279.

V3. Video showing vertical sections of PB180516.

V4. Video showing cross sections of PB180516.
